# “One stop” clinic for upper gastrointestinal cancer—an alternative to “straight to test” referrals?

**DOI:** 10.1007/s11845-021-02647-7

**Published:** 2021-07-20

**Authors:** Marina Yiasemidou, Ross Lathan, Manfred Lambertz, Chitakattil Oommen, Ian Chetter

**Affiliations:** 1 Rotherham General Hospital, South Yorkshire Rotherham, UK; 2 Hull York Medical School, University of Hull, HU3 2JZ Hull, UK; 3 Bradford Teaching Hospitals, BD9 6RJ Bradford, UK

**Keywords:** Early diagnosis, Endoscopy, Gastric cancer, Oesophageal cancer, Outpatient clinic

## Abstract

**Background:**

Patients suspected to have upper gastrointestinal (UGI) cancer can be referred directly for investigation; however, at times this may result to inappropriate referrals. This study explores the model of a “one-stop” clinic as an alternative to the direct referral system. The current study aims to assess the feasibility and outcomes of a one-stop UGI clinic and evaluate sensitivity and specificity of “on-the-day” diagnoses.

**Methods:**

A retrospective analysis of case notes of patients seen in one-stop clinic, between January 2017 and January 2019, was conducted. All General Practitioner (GP) referrals were screened by a specialist nurse.

**Results:**

After completion of the post-GP referral screening process, 252 patients (median age 68 years, IQR 58.8–77.3 years; M:F ratio 118:134) were allocated to the one-stop clinic. OGD was not required, contra-indicated or declined in 27 cases (10.7%). The records of three patients could not be found. One patient did not attend. Overall, 221 patients underwent testing and received “on-the-day” diagnoses. Sensitivity was 94% (range 87–100%), and specificity was 92% (88–96%). Ninety-six percent of patients received a diagnosis on the day.

**Conclusions:**

The one-stop clinic was feasible and had good specificity and sensitivity. The finding of 10.7% of cases not being suitable for OGD indicates that a patient/specialist consultation is necessary to prevent misuse of endoscopy appointments. The authors recommend widespread adoption of one-stop clinics in UGI surgery.

## Introduction

Every year, a significant number of patients are referred to hospital by their GP (general practitioner) to be investigated for upper gastrointestinal cancer (UGI). In 2017–2018 alone, 185,279 patients were referred to a hospital in the UK for that purpose. The current operational standard for assessment in the 2-week wait pathway is 93%. In 2018, UGI was the only cancer cohort to not meet this target [1], raising the question as to whether changes should be made to the current service provided, in order to increase efficiency. Moreover, as emphasised in the NHS England 10-year plan, reducing the time to diagnosis is instrumental for improving overall cancer survival rates [2, 3]. It is therefore imperative to expedite time to diagnosis.

Patients referred through the urgent cancer pathway are subject to certain time targets. These include waiting no more than 62 days from GP referral and no more than 31 days from their diagnosis to their first definitive treatment [4, 5]. Therefore, rapid diagnosis is crucial as the time taken for referral to a tertiary centre for treatment is added to the overall wait from diagnosis to treatment. This is particularly important for gastrointestinal cancer, where reduction of endoscopy waiting times is associated with an increase in resection rates [6]**.**

Currently, the common practice in many trusts includes an initial specialist review which is followed by investigations that are not necessarily performed on the same day [5, 7]. Some trusts may operate “straight to test” policies whereby the patient is referred by their GP for endoscopy [8]; however, if endoscopy lists are saturated or further information is needed prior to testing, the patients are seen in the outpatient clinic prior to having a diagnostic test performed [8]. Moreover, there have been reports that a significant percentage of patients referred through the 2-week pathway straight to test were unsuitable or failed a telephone assessment. In lower gastrointestinal surgery, this was combated by an additional screening process by dedicated lower GI (gastrointestinal) specialists for colorectal cancer [8]. These results indicate that the lack of a screening process after GP referral may result in many inappropriate appointments and waste of resources that could be utilised in a more efficient way.

A suggested way to speed up diagnoses of cancer and other pathology are “one-stop” clinics [9–11]. Although one-stop clinics were pioneered successfully in breast cancer surgery [10, 12–16], it is a model that has been used successfully in other specialties as well [9, 11]. Typically a patient attends the one-stop clinic and has investigations and a diagnosis within the premise of the clinic, all performed within one visit [9–11]. One-stop clinics demonstrated excellent sensitivity and specificity in diagnosing cancer, whilst being cost-effective [10] and reducing the need for admission to hospital [9]. The benefits for patients were undeniable: reduced number of hospital visits, discussions and management by one doctor (or one team) with whom the patient can build a rapport and most importantly patient reassurance through reduced time to reach diagnosis [9].

Whilst one-stop clinics are appealing both to patients and doctors [17], their application in some specialties may be more tasking than others [9]. For instance, the application of a one-stop clinic in upper gastrointestinal surgery would require the introduction of endoscopy and as a result patients would require prior preparation, organisational restructuring and consultation [9]. Due to the above, some authors question the feasibility and efficiency of one-stop clinics in UGI surgery [9].

Here we present the successful application of a one-stop clinic for gastrointestinal cancer. The primary aim of this study is to assess feasibility of a one stop UGI cancer clinic. Secondary aims include calculating the sensitivity, specificity and percentage of patients given a diagnosis on the day and percentage of patients necessitating an OGD.

## Methodology

This project was approved by the trust’s Research, Innovation and Clinical Effectiveness department (reference SE 0747).

A retrospective analysis of patient case notes seen in a ‘one-stop’ UGI cancer referral clinic between January 2017 and January 2019 was performed. Follow-up ranged from 6 to 24 months. Using a bespoke data collection tool, information on patient demographics (age, gender), endoscopic findings, clinic diagnosis and consolidated diagnosis were collected. A consolidated diagnosis (of normal/benign or malignant) was reached after imaging, histology or further testing (e.g. CT scan). If there was primary malignancy in any region other than the upper GI tract (oesophageal and gastric), the consolidated diagnosis for the purposes of assessing the one-stop UGI clinic would be normal or benign, as the malignancy was not in the UGI tract and therefore could not possibly be detected during the UGI clinic.

Prior to clinic attendance, patients referred from GP were screened by the UGI specialist nurse. This process involved review of the paperwork sent by the GP and a further phone conversation with the GP surgery when that was deemed appropriate. Patients were then triaged to the “one-stop clinic”, or to a traditional 2-week wait clinic. Although there were no strict criteria for diversion to the one-stop clinic, the GI specialist nurse was aiming to identify the patients most likely to require endoscopic evaluation. This process included finding a balance between indications and patient fitness.

The one-stop clinic consisted of a consultation and an investigation session (i.e. endoscopy). The morning former involved history taking and clinical examination by a consultant surgeon. The patients found to require an OGD investigation after counselling at the first session (as well as the GP practice, as part of the 2-week referral process) proceeded to having this after informed consent was obtained, during the afternoon session. A second consultation took place after the endoscopy session giving the patient an “on-the-day” diagnosis. A further communication (via a written letter addressed to the patient and GP or invite to clinic) was then arranged to inform the patient and their GP of the consolidated diagnosis and potential further diagnostic tests or treatment.

For purposes of data analyses, the on-the-day clinic diagnoses were grouped as malignant, suspicious, atypical, benign and normal. This categorisation was based on similar analysis done for breast surgery clinics [10]. Barrett’s oesophagus was categorised as atypical to distinguish it from diagnoses that are benign without the potential for malignant progression (e.g. hiatus hernia or oesophagitis). Consolidated diagnoses were grouped as malignant or normal/benign. This categorisation allowed for statistical analysis establishing the sensitivity and specificity of the one-stop clinic.

Sensitivity was calculated as true malignancies (malignant consolidated diagnosis) divided by the sum of true malignancies and false benign (i.e. benign diagnosis on the day but malignant consolidated diagnosis). Specificity was calculated as true benign cases (benign consolidated diagnosis) divided by the sum of true benign and false malignant (i.e. malignant diagnosis on the day but benign consolidated diagnosis).

In order to process the two-by-two table for the sensitivity and specificity analysis, on-the-day diagnoses were grouped in clinically appropriate groups. Analyses were performed using all such combinations (Appendix), resulting in a range and mean for sensitivity and specificity. Descriptive statistics and plots were completed using JASP 10.13 statistical open source software [18].

Simple descriptive statistics were used to calculate all other percentages.

## Results

### Suitability for OGD

After completion of the GP referral screening process, 252 patients (median age, 1st–3rd IQ: 68, 58.75–77.25, M:F 118:134) were allocated to the one-stop clinic. All other GP referrals were allocated to a traditional 2-week wait clinic. Nine patients were excluded from further analysis, as during the morning consultation it was established that an OGD was not indicated. Eighteen patients did not have an OGD, either because they refused the investigation, they had a contraindication for this to be done on the day (e.g. not nil by mouth) or they required a colonoscopy and an OGD to further investigate their symptoms and this could not be offered on the specific clinic. Therefore, 27 patients were unsuitable for OGD (10.7%).

### Sensitivity and specificity

**Fig. 1:**
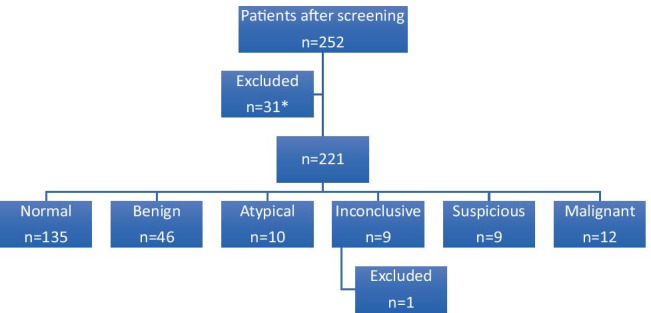
“On-the-day” diagnosis. *n = 9: OGD not clinically indicated, *n* = 10: declined OGD, *n* = 4 contraindication for OGD on the day e.g. not nil by mouth, *n* = 4 required both colonoscopy and OGD, *n* = 3 record of follow-up not found, *n* = 1 patient did not attend

The follow-up records of three patients could not be found and therefore consolidated diagnosis could not be established; as a result, these were excluded from further analysis. One patient did not attend. Overall, 221/252 patients underwent testing and received “on-the-day” diagnoses. One patient, whose OGD was inconclusive, refused any further follow-up, alas not allowing for a consolidated diagnosis; their case was excluded from further analysis (Fig. 1).

**Fig. 2:**
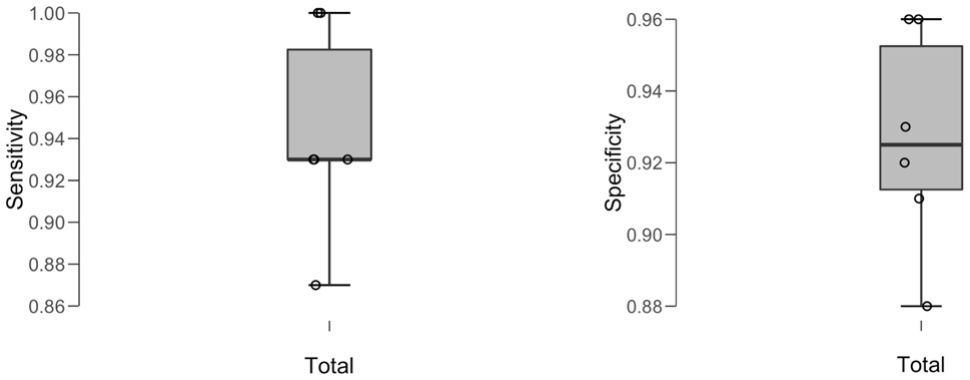
Top left: sensitivity distribution, top right: specificity distribution

After clinically appropriate grouping of the ‘on-the-day’ diagnoses (please see Appendix), mean sensitivity for the one-stop UGI clinic was 94% (range 87–100%) and specificity was 92% (88–96%) (Table 1). Unsurprisingly, higher sensitivity occurred in the expense of specificity as shown in Fig. 2.

**Table 1:** Descriptive statistics of sensitivity and specificity value

		Sensitivity		Specificity	
Mean		0.943		0.927	
Std. deviation		0.050		0.031	
Minimum		0.870		0.880	
Maximum		1.000		0.960	
25th percentile		0.930		0.912	
50th percentile		0.930		0.925	
75th percentile		0.983		0.953

### On-the-day diagnosis

On-the-day diagnosis was 96% i.e. *n* = 9 inconclusive diagnosis.

## Discussion

One-stop UGI clinic is feasible with 96% of patients being provided with an “on-the-day” diagnosis. This may alleviate patient anxiety and reduce hospital visits/admissions. The clinic was associated with an excellent sensitivity and specificity in detecting upper gastrointestinal malignancy. Although referrals underwent a rigorous pre-screening process, 10.7% of the patients who were allocated to the one-stop clinic did not require, declined or had contraindications for an OGD on the day of the appointment. Had these patients been referred through the “straight to test” process, the endoscopy appointments would have been underutilised.

The results of the current study support findings of similar studies assessing one-stop clinics in other specialties [10]. One-stop cancer clinics have been shown to be associated with significant reduction in interval time to testing, and increased percentage of patients to receive an on the day diagnosis ^5–6^. Delaloge et al*.* [10] performed retrospective diagnostic accuracy analysis of a one-stop breast cancer clinic ^4^. Using similar methods to our study, lesions were grouped into malignant and benign with two further groups of suspect/atypical and undetermined. Results were remarkably similar to our study with sensitivity, specificity and “on the day diagnosis” rates of 98.4%, 99.8% and 75% respectively [10]. It is noted that the “on the day diagnosis” is somewhat dissimilar to the one quoted for the current study. This may be because GI lesions, unlike breast ones, can be directly visualised by an experienced clinician. Moreover, modern endoscopic technologies, through which the architectural pattern disruption of polyps can be assessed, may have contributed to giving a more definitive answer to the patients on whether the lesion is malignant or not.

In addition to the diagnostic value, a randomised controlled trial, allocating 670 women to a dedicated breast cancer surgery clinic or a one-stop clinic, found one-stop clinic attendance to be associated with significantly reduced anxiety [17].

Although it is beyond the scope of the one-stop UGI clinic, not being able to provide an answer on whether there is malignancy in the parts of the gastrointestinal tract that cannot be inspected by an OGD, one may argue that patient anxiety cannot be completely alleviated by this clinic. This is particularly true for patients that present with loss of weight and will require to have a CT scan (computed tomography) to further investigate this. Unlike, the breast surgery one-stop [17], there is no imaging involved in the UGI clinic described here. One-stop breast clinics use mammogram and ultrasound scan as their imaging modalities [19–24], which admittedly, are less resource and time consuming than performing and reporting a CT within the time confinements of a one-stop clinic. It should be noted that after the completion of this study, staging scans have been introduced in one-stop UGI clinic for patients with a malignant diagnosis. This is aimed to expedite referral to a tertiary centre and treatment.

In addition to one-stop clinics, a number of diagnostic testing pathways have been explored. Direct access testing (e.g. CT/MRI) has also been studied as a route to reduce time to diagnosis [25–28]. These models have been successful, showing reduction in time to treat [25, 26]. However, they have been compared with the traditional 2-week wait clinics which involve multiple attendances to hospital [25, 26]. Assessment of time to treat was beyond the scope of this study but would be interesting for future comparisons to be made between the straight-to-test model and one-stop clinics.

It is of note that despite the rigorous pre-screening process by a UGI specialist nurse, 10.7% of the patients did not meet the criteria, declined or were not prepared for an OGD on the day. This finding may indicate that a direct patient/specialist consultation is necessitated, in order to minimise the misused endoscopy appointments. These can be in the form of face-to-face or virtual consultations; the later have been in the spotlight in the past weeks due to the rapid increase in usage during the COVID pandemic [29–31]. Some authors advocate their introduction to healthcare even after the resolution of the COVID-19 crisis [32] and it is something that should be explored in the future for one-stop clinics.

Although both straight-to-test and one-stop clinics have shown good results and are popular with patients and doctors [9, 11, 12, 14–17, 20], the results of the current study demonstrate that one-stop clinics provide the benefit or swift diagnoses whilst eliminating the underutilisation of endoscopy appointments. It is for that reason that the authors of this study cautiously recommend the widespread adoption of one-stop UGI clinics.

## Appendix. Sensitivity and specificity two-by-two tables

**Table:**

Clinic diagnosis	Consolidated diagnosis		
	Malignant	Normal/benign	Total
Malignant/ Suspicious/ Atypical	14	17	31
Normal/benign	0	181	181
Total	14	198	212

**Table:**

Clinic diagnosis	Consolidated diagnosis		
	Malignant	Normal/benign	Total
Malignant/suspicious	13	8	21
Atypical/ Normal/ Benign	1	190	191
Total	14	198	212

**Table:**

Clinic diagnosis	Consolidated diagnosis		
	Malignant	Normal/benign	Total
Malignant/ Suspicious/ Atypical/ Inconclusive	15	24	39
Normal/benign	0	181	181
Total	15	205	220

**Table:**

Clinic diagnosis	Consolidated diagnosis		
	Malignant	Normal/benign	Total
Malignant/ Suspicious/ Atypical	14	17	31
Benign/ Normal/ Inconclusive	1	188	189
Total	15	205	220

**Table:**

Clinic diagnosis	Consolidated diagnosis		
	Malignant	Normal/Benign	Total
Malignant/ Suspicious/ Inconclusive	14	15	29
Atypical/ Normal/ Benign	1	190	191
Total	15	205	220

**Table:**

Clinic diagnosis	Consolidated diagnosis		
	Malignant	Normal/benign	Total
Malignant/ Suspicious	13	8	21
Atypical/ Normal/ Benign/ Inconclusive	2	197	199
Total	15	205	220
